# Public Health Entrepreneurship: A Novel Path for Training Future Public Health Professionals

**DOI:** 10.3389/fpubh.2019.00089

**Published:** 2019-04-24

**Authors:** Elisabeth R. B. Becker, Teresa Chahine, Ross Shegog

**Affiliations:** ^1^Department of Health Promotion and Behavioral Sciences, School of Public Health, University of Texas Health Science Center, Houston, TX, United States; ^2^Yale School of Management, New Haven, CT, United States

**Keywords:** entrepreneurship, innovation, twenty first century skills, public health education for professionals, training

## Abstract

**Background:** As schools of public health adapt to the new Council on Public Health (CEPH) competencies there is increased relevance in training public health professionals in public health entrepreneurship. Public health entrepreneurship provides an alternate process to traditional academic approaches focusing on translating public health knowledge into effective, sustainable, and scalable solutions.

**Objective:** This study reports student perceptions of public health entrepreneurship and training needs for successfully equipping future public health professionals.

**Methods:** Focus groups were conducted in April 2018 with graduate public health students in pilot entrepreneurship courses at two U.S.-based CEPH-accredited schools of public health.

**Results:** Participating students (*n* = 29) were mainly pursing MPH degrees (62%) within Health Management and Policy (38%) or Health Promotion/Global Health (31%) departments. Most students (52%) were between 21 and 30 years old. For 71% of students this was their first academic course with a focus on entrepreneurial thinking. Four themes emerged regarding public health entrepreneurship and training needs for becoming a successful public health professional of the future. Students confirmed a place for public health entrepreneurship in the emerging educational paradigm because it is action-oriented, skills-driven, and fosters innovation through inter-professional collaboration and cross-pollination of knowledge and skills between professional disciplines.

**Conclusions:** The competencies required for public health entrepreneurship are in alignment with CEPH competencies and are well-received by the next generation of public health professionals as an adjunct but nascent approach to stimulate public health innovation.

## Introduction

Over the past century public health has evolved from a historically “siloed” and research-centric field to one that is more interdisciplinary, inter-professional, and action-centric (1–3). To better prepare the future public health workforce, the Council on Public Health Education (CEPH) began implementing in 2016 its largest accreditation change in over 40 years. These accreditation changes introduced a move toward greater accountability and measurement, competency-based skill training, and marketability for new Masters of Public Health and Doctors of Public Health graduates (4). A wealth of knowledge was collected as a result of the historical focus on research to understand causes and pathways of disease at the population level. More recent efforts call for a translational focus in public health to apply that knowledge base toward action and solution implementation that are sustainable over time (5–7). Additionally, there is an increased recognition that a translational focus that addresses the social and environmental determinants of health and incorporates collaborative expertise on poverty, gender, housing, transportation, climate, and humanitarian challenges among others is of paramount importance (3, 8, 9).

In this context, the discipline of entrepreneurial-driven public health innovation has emerged as an approach to create novel interventions, products, and services that address public health problems (8–12). Public health entrepreneurship (PHE) is the application of entrepreneurial skills to advance the public health mission (13). The entrepreneurial framework holds promise as an extension to traditional approaches for enhancing reach and providing fiscal models to sustain public health interventions, products, and services that today rely heavily on government or donor funding (12). As universities adapt to the new CEPH competencies, there may be added advantages in increasing pedagogical offerings in PHE. As pilot programs in PHE are emerging it is important to explore how such offerings are perceived by the public health students they are designed to serve.

The purpose of this study is to describe the perceptions of graduate public health students regarding PHE and their training needs for becoming successful public health professionals of the future. To our knowledge this is the first research study examining perspectives of PHE in the academic setting and incorporating student opinions (14, 15).

## Materials and Methods

### Course Descriptions

Students participating in this study were enrolled in one of two courses either the Harvard T.H. Chan School of Public Health (HSPH), Course HPM557: Innovation and Entrepreneurship in Health Care, or the University of Texas School of Public Health (UTSPH), Course PH1498: Technology, Entrepreneurship and Applied Innovation in Public Health.

#### HPM557: Innovation and Entrepreneurship in Health Care

Seventy-eight students were enrolled in HPM557. The course, in its third year, took place over 21-h in the fall semester. HPM557 utilized the case method, with professionals often joining the case discussion as guest speakers, alongside in-class exercises for student teams. The course was designed to expose students to the theory and practice of innovation and entrepreneurship in health care settings. The course required building skills in three domains. (1) Understanding the entrepreneurial process of building an early stage health care startup venture (non-profit or for-profit) including developing an idea and its value proposition; creating a business model; developing a plan for financing and execution; deciding on and recruiting a team with appropriate skillsets; evaluating the structure and function of the venture and proposing improvements. (2) Conducting a needs assessment including evaluating the “innovation environment”; identifying barriers and facilitators to adoption and implementation for innovation in any established health care organization; and writing a proposal for recommendations for improvement. (3) Securing partnerships and investment including developing persuasive pitches for ventures that provide sustainable solutions to public health problems. The course concluded with students delivering an elevator pitch for an innovation-driven startup idea.

After the conclusion of the course, eighteen students continued to work on their startup ideas through a pilot program initiated in spring 2018. The pilot program provided financial and mentorship support to student teams participating in university-wide entrepreneurship competitions. Participants (*n* = 44) included 18 HSPH students from HPM 557 in addition to 13 HSPH students who had not taken the course and 11 other individuals from outside of HSPH who had been recruited to join the student teams.

#### PH1498: Technology, Entrepreneurship, and Applied Innovation in Public Health

Nineteen students were enrolled in PH1498. The course was a 15-h pilot seminar initiated in spring 2018. PH1498 featured guest speakers with knowledge in public health entrepreneurship and venture creation. The course was designed to increase student awareness of the application of entrepreneurial thinking in creating startups and social ventures that address public health challenges. Learning objectives were to describe: (1) Emerging technology trends impacting public health; (2) social entrepreneurship and its relation to public health, business and other disciplines; (3) skill-sets and perspectives for public health professionals to thrive in non-traditional public health careers; (4) innovative ways to fund social ventures. The course concluded with students delivering an elevator pitch for an innovation-driven startup idea.

### Study Design and Sample

#### Participant Eligibility for Focus Groups

Students in the HSPH pilot program and the UTSPH pilot course were invited to participate in an extracurricular 1-h focus group in April 2018. The purpose of the focus group was to explore student perceptions about entrepreneurship and their views on training to equip the public health professional of the future. The focus group was not related to course participation or grading. The study was approved by Institutional Review Boards at the Harvard T.H. Chan School of Public Health and the University of Texas Health Science Center at Houston.

#### Measurement

The students were provided a 9-item demographic survey and a 10-item entrepreneurial orientation survey during the focus group (16). Focus groups were audio recorded. Students who consented to the study but were unable to attend the focus group were invited to provide written responses to the focus group questions (Table 1).

**Table 1:** Focus group questions.

(1) Why did you choose a career in public health? (2) Why were you interested in taking this course? (3) What did you learn from this course if anything? (4) Did your perception of public health change during the course? Explain. (5) Do you think the current public health curriculum is sufficient to address this century's public health problems? (6) Do you think that technology and entrepreneurship will help you achieve your public health goals? (7) Are there public health problems that could or should be addressed with an entrepreneurial lens? Please explain. (8) What problems would you be most likely to address with an entrepreneurial lens? (9) What could the school do to support you in your entrepreneurial or technology-driven pursuits? (10) What training and/or resources are needed for a twenty first century public health entrepreneur/technology innovator?

#### Analysis

Focus group audio recordings were transcribed verbatim. Focus group transcripts, notes, and written responses were first reviewed separately to identify themes, then combined and reviewed to ensure that patterns were consistent across format and site, in accordance with documented standard qualitative techniques (17–20). Demographic surveys were analyzed descriptively.

## Results

### Participant Profiles

Twenty-nine students (17 from HSPH and 12 from UTSPH) participated (Table 2) with 21 providing in-person feedback and 8 providing their responses in a written format within 3 weeks of the focus group. Most students were pursuing MPH (62%) or DrPH (24%) degrees, were from departments of Health Management and Policy (38%) or Health Promotion/Global Health (31%), were 21–30 years of age (52%), and were taking their first academic course with a focus on entrepreneurial thinking (71%). The top three expected work settings (non-exclusive) after graduation included NGO/non-profit/foundation (69%), clinical/health care (62%), and a startup venture (52%). Most students reported preferences for risk-taking (71%), innovative thinking (69%), and proactive problem solving (82%) consistent with an entrepreneurial orientation (16).

**Table 2:** Participant demographic survey responses.

	**HSPH (*n* = 17)**	**UTSPH (*n* = 12)**	**Total (*n* = 29)**
	***n***	***n***	***n*(%)**
**Academic departments**			
Health Management and Policy	7	4	11 (38)
Health Promotion, Behavioral Science, Global Health	3	6	9 (31)
Epidemiology, Quantitative methods	2	2	4 (14)
Interdisciplinary^*^	5	–	5 (17)
**Programs**			
MPH	10	8	18 (62)
DrPH	5	2	7 (24)
MS/SM	2	–	2 (7)
PhD	–	2	2 (7)
**Year in program**			
1	11	1	12 (41)
2	6	5	11 (38)
3^+^	–	6	6 (21)
**International student**			
Yes	9	3	12 (41)
No	8	9	17 (59)
**Gender**			
Female	5	10	15 (52)
Male	12	2	14 (48)
**Age range**			
21–30	11	4	15 (52)
31–40	4	4	8 (2)
41^+^	2	4	6 (21)
**First academic entrepreneurial course?^∧^**			
Yes	9	11	20 (71)
No	7	1	8 (29)
**Expected future work setting^+^**			
NGO/non-profit/foundation	9	11	20 (69)
Clinical/health care (i.e., hospital, community clinic)	8	10	18 (62)
Startup	10	5	15 (52)
Industry (i.e., large for-profit-corporation)	11	3	14 (48)
Government (i.e., health department)	3	9	12 (41)
Technology sector (i.e., Google)	11	1	12 (41)
Academics (i.e., research, teaching)	3	5	8 (28)
Entrepreneurial orientation^**^	%	%	%
Risk	80	61	71
Innovation	73	64	69
Proactivity	77	89	82

* *HSPH DrPH students are not affiliated with an academic department*.

∧ *1 student omitted a response to this survey question*.

+ *Survey question, “How likely are you to work in the following setting?” was answered on a 5-point Likert scale. Above data represents those that responded “somewhat likely” or “very likely.” Categories are non-exclusive*.

** *Percent agreement on 5-point Likert scale with response ratings from strongly disagree to strongly agree*.

### Thematic Analysis

Four themes regarding students' perceptions of the current academic public health paradigm, PHE, and future training needs emerged. These themes are described below and summarized in Table 3.

**Table 3:** Emergent themes.

1. Public health research must be accompanied by action.
2. Public health entrepreneurship provides a potential pathway for action.
3. Public health entrepreneurship requires a unique skillset to advance public health.
4. Public health entrepreneurship presents an opportunity for inter-professional collaboration and cross-pollination of knowledge across disciplines.

#### Theme 1: Public Health Research Must Be Accompanied by Action

Students expressed that while the public health curriculum is preparing them to analyze public health problems, they need more training in solution implementation. Student perceptions indicated the need for more “real world” training to bridge the gap between the current knowledge base and the actions public health professionals are collectively taking to apply that knowledge to deliver effective, sustainable, and scalable solutions.

Students spoke of their fatigue with describing public health problems and collecting data without solving the problems in timely and sustainable ways. They agreed that research is a necessary antecedent for improved understanding of causal factors and their empirical association with public health problems. They also agreed that, despite this improved understanding of the problem, efforts to solve the problem appeared secondary to explaining it, and that proposed solutions were often not sustainable in the long term. Students expressed frustration with the focus of faculty members on publishing papers on the same topic rather than forging connections and collaborations with professionals to translate their results into action.

“*I was just sick and tired of describing the problem. I had just had it, oh really there's more health disparities fantastic. I can't take anymore description of the problem. There's got to be solutions and interventions that are done. Whether it be tech based or not, that was the avenue I started going down.”*

- UTSPH (auditing), recent HSPH MPH grad, Health Policy, female

“*Less data more action. We sit around wanting to prove obvious solutions instead of trying anything out ourselves. I most certainly do not think the current public health curriculum is sufficient to address this century's public health problems.”*

- HSPH, MPH program, Global Health, male

#### Theme 2: Public Health Entrepreneurship Provides a Potential Pathway for Action

Students discussed how applying an entrepreneurial approach to public health problems offered an alternative way to develop solutions for those problems. For many students this was their first study of how practitioners in the commercial sector develop and operate non-profit and for-profit startup ventures that have a public health focus. They viewed PHE as an opportunity to challenge conventional thinking and act boldly without the constraints imposed by an academic research methodology, stoke innovation by adopting knowledge from commercial sectors outside of public health academia (cross-pollination), and to use a business model as a means to financially sustain solutions beyond traditional grant mechanisms.

“*Innovation and entrepreneurship are driving forces for change being largely neglected in the field of public health. I am convinced that entrepreneurship, innovation, management and leadership are very important skill sets in public health. Current public health curricula are largely tied in with academia and it is not sufficient to address all the public health problems*.

- HSPH, DrPH program, interdisciplinary, male

The students also saw a dual-value in PHE training because entrepreneurship is both a mindset and a skillset. An entrepreneurial mindset cultivates the creative confidence to imagine solutions and the entrepreneurial skillset allows students to systematically test and iterate the steps and resources needed to build, launch, and implement their solutions.

“*For me entrepreneurship is a state of mind, it's about connecting things that are not connected today. If you have an entrepreneurial spirit you can see “oh, maybe this can go with this.” Like taking the example of what Uber is doing with some hospitals bringing Uber expertise to bring patients to hospital to reduce the no-show rate, that's what I think entrepreneurship is. If you are exposed to entrepreneurial thinking then you start connecting and saying “ah yeah maybe I have to look at these things this way.”*

- UTSPH, PhD program, Management, Policy and Community Health, male

#### Theme 3: Public Health Entrepreneurship Requires a Unique Skillset to Advance Public Health

Students indicated a need for skill-building and support related to interdisciplinary team formation and collaboration; financing public health innovations; network building, and organizational management.

“*[A 21st century public health entrepreneur will need training in] strengthening our “elevator pitch skills,” learning more about the differences in company development i.e. the nuts and bolts of choosing to develop a 503-c vs. an LLC etc…, and then how to actually set up a company, which paperwork needs to be filed, how much does it cost, establishing realistic timelines. Developing an idea is not necessarily the difficult part but really learning about how to go forward with the idea requires a wider knowledge base.”*

- UTSPH, DrPH program, Management, Policy and Community Health, female

Students also emphasized that in addition to launching new interventions, products and services, PHE is needed within existing public health institutions (“intrapreneurship”). Students were careful to point out that entrepreneurship in the public health setting may differ from other forms of entrepreneurship, which can result in increasing social disparities or increasing healthcare costs without delivering improved health outcomes.

“*The high rising cost of health expenditure was a result of entrepreneurial spirit under unbalanced information and competition. I am looking forward to seeing more disruptive innovation in the fields of healthcare delivery to solve that problem.”*

- HSPH, DrPH program, interdisciplinary, male

Students overall expressed optimistic views of utilizing entrepreneurship to address public health. However, students recognized that PHE is not yet part of “mainstream” thinking or public health training and requires the cultivation of a unique skillset to imagine solutions and mobilize resources that advance public good that are not universally available at schools of public health. Students emphasized the importance of ensuring that entrepreneurship is integrated in a way that strengthens the tenets and values of public health rather than undermines its fundamental purpose.

“*I think any time you examine a problem from different angles and try to develop new ways to improve the issue, you are an entrepreneur. Technology can be useful but we need to pay attention to who has access and how does it improve or change the system.”*

- UTSPH, DrPH program, Management, Policy and Community Health, female

#### Theme 4: Public Health Entrepreneurship Presents an Opportunity for Inter-professional Collaboration and Cross-Pollination of Knowledge Across Disciplines

Students indicated that regardless of the role or sector they wanted to work in after graduation, an entrepreneurial mindset and skillset would be important in helping them accomplish their public health goals. They valued the potential of entrepreneurial thinking to accelerate partnerships with commercial disciplines that are often not included in the traditional academic enterprise and enable the transfer of knowledge and practices from these other disciplines in the service of improving public health. For many students these courses were their first exposure to entrepreneurial inter-professional collaborations outside an academic framework.

“*I learned how public health plays a role in the business sector, and how beneficial it is to be able to think outside the box. This course has also inspired me to read more about technology, and to keep up to date on all industries not just those traditionally thought of as public health related.”*

- UTSPH, MPH, Health Promotion and Behavioral Sciences, female

Students were continually frustrated with their programs and schools being “siloed” and disconnected from the larger innovation ecosystems at their institution and in their communities. When students in the innovation pilot at HSPH were asked what areas they needed the most support in, they responded that their primary needs were 2-fold: forming interdisciplinary teams, and securing funding. Student teams experienced difficulty recruiting members with skills in engineering and computer programming. They also voiced frustration with the time and cost incurred in commuting back and forth between the medical/public health campus and the main campus where the Harvard Innovation Labs (iLab) are located. Students from UTSPH expressed similar challenges:

“*It's amazing how many things are happening right here that you have no idea about because there is no connection, transparency, exposure. This course allows you to know what else is going on in this space in this community and that is hugely helpful. You get lots of cross-intuitional ideas. I think it's really eye opening in that way. The best thing the school can do is show you the other things that are happening in the Texas Medical Center, to open up avenues, opportunities for you to work across disciplines and work across intuitions.”*

- UTSPH (auditing), recent HSPH MPH grad, Health Policy, female

Students discussed opportunities for current academic public health curricula to expand to include courses on entrepreneurship and the desire to collaborate on projects and leverage existing resources to address real world public health challenges with peers and professionals from different domains.

“*I chose a career in public health because it's a diverse, interdisciplinary field addressing pressing local and global problems. Public health is everywhere. Public health has a lot of potential for innovation. I wish we had more entrepreneurship and innovation projects, courses, seminars, etc. It feels like we're isolated from iLab, SEAS, MIT, etc. I want to meet and work in teams across different programs and departments and create real world projects… but the school seems quite siloed-geographically and academically.”*

- HSPH, DrPH program, interdisciplinary, female

“*One of the things that attracted me to public health was that is very diverse and it can take you down various different paths. I feel the curriculum is lacking courses that highlight opportunities in the private sector. While government and non-profit play a huge role and are important in addressing many public health problems, we often forget to look elsewhere for innovative solutions. Approaching problems from as many perspectives as you can, will help lead to better solutions… the most important take away is not to restrict ourselves to any one way of thinking.”*

- UTSPH, MPH, Health Promotion and Behavioral Sciences, female

## Discussion

This study shares the results of pilot offerings introducing PHE into the curriculum. These courses are consistent with the CEPH competencies that call for active, integrated, student-centered approaches. Such courses may stimulate the use of creative strategies and experimentation within schools and programs of public health across the U.S. (2, 5, 9, 14, 15). This study is the first to examine student perceptions on PHE and to provide feedback on training and resource needs to advance pedagogy and practice in this domain.

These results emphasize the sense of priority felt by incoming public health trainees to apply the wealth of knowledge collected to date by the public health community, and translate this knowledge into action (3, 6). The new CEPH competencies, focused on skill-based criteria, attend to student objectives to acquire marketable skillsets. In this vain, this study demonstrates that students also have an appetite for the PHE skillset. PHE provides an opportunity to combine the strengths of current public health training with new and existing approaches of social innovation and entrepreneurship (10, 12). PHE reflects both the mindset of questioning assumptions, challenging the status quo, and imagining bold new solutions; and the skillset of creating new interventions, products and services to address public health needs. Advantages of PHE include that it: (1) is consistent with existing interventions and community action models in delineating the design, prototyping, implementation, evaluation, and scaling of evidence-based solutions in both new and existing settings; (2) is a vehicle for expanding the public health workforce and the scope of resources available to achieve improved public health; (3, 7, 21); and (3) offers the potential to effectively engage with the myriad stakeholders involved in public health beyond sectors and institutions traditionally associated with public health.

However, PHE is a nascent field which requires implementation within future public health curricula before it can be fully emergent. Research and evaluation to define the key characteristics and core competencies of successful training in PHE will be necessary to inform improvements in pedagogy and practice. New indicators of success are needed to determine whether and how PHE training impacts participants in their future careers. Such metrics might include measures of career success of graduates (i.e., job position, longevity, patents and copyrights, and earnings); measures of impact on public health (i.e., reach, dissemination, and cost-benefits accrued from products and services); and measures of sustainability (i.e., long-term viability of products and services).

Limitations of this study include a small sample of self-selected students who may not reflect the viewpoint of the broader student body. Further, the sample was drawn from classes in only two universities and is not inclusive of all universities with PHE in their curricula (8). Despite this, these findings can inform other schools and programs of public health who are introducing PHE into their curriculum.

Accredited schools of public health were required, by December 2018, to provide skills training to build student competencies in systems thinking, intervention design, evaluation, resource allocation, stakeholder mobilization, and collaboration; and capabilities in leading organizational change, strategic planning, generating revenue, working in inter-professional teams, and communicating across sectors and levels of stakeholders (4). PHE may provide new pathways to mobilize resources and stakeholders to tackle twenty first century public health challenges across disciplines and sectors that align with these updated CEPH competencies.

## Ethics Statement

This study was approved by Institutional Review Boards at the Harvard T.H. Chan School of Public Health and the University of Texas Health Science Center at Houston. All subjects gave written informed consent in accordance with the Declaration of Helsinki.

## Author Contributions

EB, TC, and RS contributed to study conception and design, data collection and interpretation, manuscript preparation and review.

### Conflict of Interest Statement

The authors declare that the research was conducted in the absence of any commercial or financial relationships that could be construed as a potential conflict of interest.
